# Prediction of treatment response by anticitrullinated protein antibodies (ACPA) levels among patients with rheumatoid arthritis

**DOI:** 10.1186/1479-5876-10-S3-P44

**Published:** 2012-11-28

**Authors:** Raquel Hernández, Paz González, Alejandro Muñoz, Jose Luis Marenco

**Affiliations:** 1 Reumatology, Hospital Virgen de Valme, Sevilla, Spain

## Background

The relationship between ACPA and the clinical outcome of rheumatoid arthritis (RA) under treatment has been evaluated in clinical trials with conflicting results. Thus, the response to treatment was influenced by ACPA in the BeSt study. However, radiological damage progression was more likely among ACPA-positive patients. Conversely, ACPA-negative patients achieved less remissions in the IMPROVED study. Because of these, we evaluated the clinical response (CR) of patients with RA receiving treatment according to ACPA titers.

## Patients and methods

All patients seen at our Unit who fulfilled the following criteria were included in this retrospective study: 1) Diagnosis of RA by a rheumatologist meeting the 1987 criteria for RA; 2) Available determinations of ACPA; 3) Treatment for AR (whether or not biological therapy) with a minimum follow up of 6 months. The outcome variable was CR defined as reaching DAS28 <1.6. Predictors of CR were evaluated using logistic regression.

## Results

71 patients were included, 79% of them women. CR was observed in 19 (27%) patients during the first 12 months of follow-up. Baseline median (IQR) ACPA levels were 306 (7-500) for individuals without CR and 76 (7-153) for those with CR (p=0.022). 29 (56%) patients without CR vs. 5 (26%) with CR showed ACPA levels ≥200 (p=0.028). ACPA levels decreased significantly among patients with CR (p=0.019), but they did not change in those without CR (p=0.330). Factors independently associated with CR were: recent onset RA [adjusted odds ratio (AOR) 0.19; 95% confidence interval (95%CI) 0.41-0.90; p=0.036], baseline ACPA levels ≥200 [AOR 6.48; 95%CI 1.12-37.3; p=0.037], baseline CRP levels [per unit increase; AOR 1.06; 95%CI 1.001-1.12; p=0.048], baseline DAS28 [per unit increase; AOR 3.04; 95%CI 1.3-7.4; p=0.014].

## Conclusions

ACPA levels can predict CR of patients with RA receiving treatment in real life conditions. Individuals with high ACPA levels may benefit from a more aggressive treatment approach. Titers of ACPA may be useful to monitor the clinical activity of RA.

